# Polyrotaxane‐Assembled Semi‐Interpenetrating Polymer Electrolytes Enabling High‐Voltage Lithium Metal Batteries

**DOI:** 10.1002/advs.202521892

**Published:** 2026-02-26

**Authors:** Tianyi Wang, Yue Ma, Jiacheng Liu, Zhaofan He, Jin Liang, Kailiang Ren, Jie Kong

**Affiliations:** ^1^ Shaanxi Key Laboratory of Macromolecular Science and Technology School of Chemistry and Chemical Engineering Northwestern Polytechnical University Xi'an P. R. China; ^2^ Center for Nano Energy Materials State Key Laboratory of Solidification Processing School of Materials Science and Engineering Northwestern Polytechnical University Xi'an P. R. China

**Keywords:** high‐voltage lithium metal battery, high‐voltage tolerance, polyrotaxane, semi‐interpenetrating polymer electrolytes, solid polymer electrolyte

## Abstract

Polyether‐based solid polymer electrolytes suffer from low ionic conductivity at room temperature, poor oxidative stability at high voltages, and susceptibility to dendrite growth, all of which hinder their application in high‐energy‐density lithium batteries. Herein, we report a polyrotaxane‐assembled supramolecular semi‐interpenetrating network electrolyte (SSNE), synthesized through in situ polymerization of polyrotaxane with crosslinked polydioxolane (PDOL). The key innovations of this architecture are threefold: it creates hierarchical ion‐conduction pathways, introduces Lewis acid sites, and imposes spatial confinement within a polyrotaxane framework. Through host‐guest interactions, these elements synergistically decouple the transport of Li^+^ ions from the migration of anions. The SSNE exhibits impressive room‐temperature ionic conductivity of 0.18 mS cm^−^^1^ and Li^+^ transference number ($t_{Li^{+}}$ > 0.73). Furthermore, fluorine‐ and boron‐functionalized polyrotaxane stabilizes the electrode–electrolyte interphase and extends the electrochemical window (> 4.9 V). The full cell prototype comprising the LiNi_0.8_Co_0.1_Mn_0.1_O_2_ cathode, the SSNE, and Li foil delivers 81.4% capacity retention after 200 cycles and rate capability up to 2C. Moreover, a 1.2 Ah pouch‐format cell operates reliably for 190 cycles at room temperature with a cutoff voltage of 4.5 V. This study integrates dynamic polyrotaxane chemistry with a semi‐interpenetrating polymer electrolyte architecture, offering a molecule‐to‐system strategy for the development of next‐generation high‐voltage lithium metal batteries.

## Graphical abstract

This work develops a polyrotaxane‐based supramolecular semi‐interpenetrating network electrolyte. Its unique architecture decouples ion transport, achieving high ionic conductivity (0.18 mS cm^−1^), a superior Li^+^ transference number (> 0.73), and an extended electrochemical window (> 4.9 V), thereby significantly enhancing the performance of high‐voltage NCM811∣∣Li full cells of polyether based.

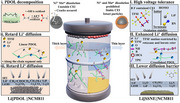

## 1 Introduction

Lithium‐metal batteries (LMBs) represent promising next‐generation energy‐storage systems due to the high theoretical specific capacity (3860 mAh g^−1^) of lithium (Li) metal anodes. This is especially advantageous for applications that require energy densities exceeding 400 Wh kg^−1^. However, the high reactivity of the lithium metal anode combined with flammable organic liquid electrolytes poses a significant safety risk [1, 2, 3, 4]. As a result, solid polymer electrolytes (SPEs) emerged as viable alternatives to liquid electrolytes, effectively addressing safety concerns such as organic solvent leakage and combustion, while enhancing interfacial compatibility with Li metal anodes [5, 6, 7, 8, 9]. Among SPEs, polyether‐based electrolytes, particularly polyethylene oxide (PEO) and poly(1,3‐dioxolane) (PDOL), are attractive due to their ether oxygen groups, which coordinate Li^+^ ions and facilitate cation hopping transport.

**SCHEME 1:**
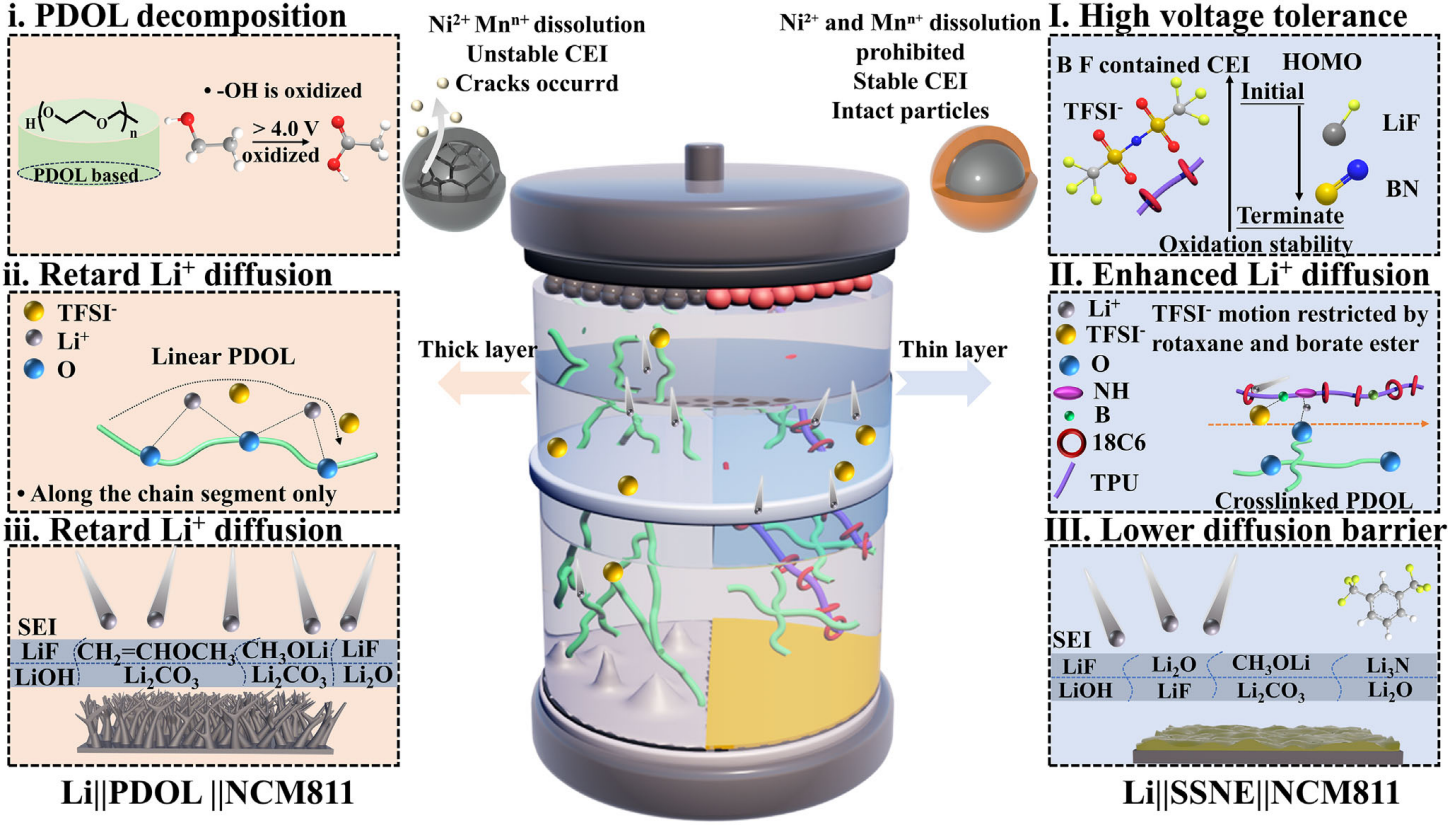
Schematic illustration of SSNE design at multiple scales. The practical issues of the Li|PDOL|NCM811 prototype, which include: (i) PDOL decomposition at high voltage; (ii) Retard Li^+^ transport along the PDOL chain; (iii) Unstable SEI and dendritic growth. The multiscale modifications for the Li|SSNE|NCM811 prototype, which include: (I) Stable CEI components enable enhanced high‐voltage tolerance; (II) Synergistic coordination accelerates Li^+^ transport while suppressing TFSI^−^ mobility. (III) The reduced Li^+^ diffusion barrier and homogeneous Li deposition barrier.

Despite their potential, the development of solid‐state lithium metal batteries (SLMBs) using polyether‐based electrolytes is hindered by inherently low ionic conductivity (< 10^−6^ S cm^−1^) and poor lithium‐ion transference number ($t_{Li^{+}}$ < 0.4) (Scheme 1ii), leading to significant concentration polarization at the anode–SPE interface [10, 11, 12, 13, 14, 15, 16]. Furthermore, the limited electrochemical stability of polyethers leads to irreversible oxidative degradation at the surface of high‐voltage cathodes (Scheme 1i) and promotes dendrite formation at Li metal anodes (Scheme 1iii). These combined limitations compromised both the achievable energy density and the cycling stability of SLMBs. Consequently, researchers have explored several strategies: (1) modification of polymer architecture or incorporating inorganic fillers (e.g., lithium aluminum titanium phosphate, Li_6.4_La_3_Zr_1.4_Ta_0.6_O_12_) to reduce crystallinity [17, 18, 19]; (2) incorporation of Lewis acids (e.g., AlF_3_, yttria‐stabilized zirconia (YSZ)), single‐ion conductors (e.g., lithium (4‐styrenesulfonyl) (trifluoromethanesulfonyl) imide, 3‐sulfonyl (trifluoromethanesulfonyl) imide propyl methacrylate), or the use of nanoconfinement to enhance Li^+^ transference number [20, 21, 22, 23, 24]; (3) fabrication of bilayer electrolytes or incorporation of functional stabilizers (e.g., LiNO_3_, CeF_3_, UiO‐66‐SO_3_Li) to improve oxidative stability [25, 26, 27, 28]; and (4) employing crosslinked polymer matrices to enhance mechanical strength and suppress dendrite formation [29, 30]. Although these strategies have led to optimization of individual metrics, comprehensively addressing the insufficient ionic conductivity, Li^+^ transference, and mechanical strength remains a formidable challenge for thin‐layer SPE membranes.

The adoption of high‐voltage cathodes such as NCM811 has enabled lithium‐metal batteries to achieve high energy densities [31, 32]. However, the poor compatibility of polyethers with high‐voltage operation (≥ 4.2 V) continues to pose a significant practical challenge [33, 34]. Enhancing the electrochemical stability of PDOL requires the development of a robust cathode‐electrolyte interface (CEI). Additives (e.g., LiBF_4_, Mg(TFSI)_2,_ and 2,4,6‐tris(4‐fluorophenyl)cyclo‐boroxine) can facilitate the in situ formation of a protective CEI layer [35, 36, 37]. Alternatively, surface‐engineered polymer coatings (e.g., polyaniline, polyimide) or inorganic layers (e.g., TiO_2_) have been proposed as artificial CEIs [38, 39, 40]. However, such a coating often increases interfacial resistance due to inhomogeneous deposition and localized degradation. Moreover, the need for precise thickness control introduces manufacturing complexity and hinders scalability. On the anode side, reactive Li deposits degrade the polymer into parasitic interfacial species with poor ionic conductivity (10^−9^–10^−11^ S cm^−1^), such as Li_2_O, ROCO_2_Li, and Li_2_CO_3_, leading to uneven Li plating and dendrite growth [41, 42]. To mitigate this issue, additives with low LUMO energies (e.g., vinylene carbonate and lithium nonafluorobutane‐1‐sulfonate) are employed to develop the sacrificial solid electrolyte interphase (SEI) [42, 43]. The incorporation of lithiophilic Li‐M alloy species (where M represents Sn, Sb, Zn, or high‐entropy alloys) further reduces the nucleation barrier and promotes high‐rate Li^+^ influx [44, 45, 46, 47]. Despite these advances, achieving a balanced strategy that simultaneously stabilizes both cathode interfacial stability and dendrite suppression at the Li anode as well as scalable manufacturability remains the key unresolved challenge for polyether‐based SPEs in high‐voltage lithium‐metal batteries.

In this work, we proposed an innovative supramolecular semi‐interpenetrating network electrolyte (SSNE) based on a polyrotaxane (PR)‐functionalized PDOL matrix. The PR structure consists of 18C6 macrocycles threaded onto thermoplastic polyurethane (TPU) backbones, forming a dynamic supramolecular network. This design spatially confines TFSI^−^, resulting in a high $t_{Li^{+}}$ of 0.73. As illustrated in Scheme 1II, the TPU‐rotaxane facilitates the generation of “free Li^+^” through multiple coordination interactions, such as C─O/Li^+^, ─C═O/Li^+^, ─NH─/Li^+^, and ─B─O/TFSI^−^, leading to enhanced ionic conductivities (0.18 mS cm^−1^ at room temperature and 0.40 mS cm^−1^ at 60°C). At the cathode, oxidation of the TPU‐rotaxane & TFSI^−^ complex forms a fluorine‐ and boron‐rich CEI, extending oxidative stability to > 4.9 V (Scheme 1I). Simultaneously, reduction of 1‐isocyanato‐3,5‐bis(trifluoromethyl)benzene (BTFB) produces a robust, LiF‐rich SEI (Scheme 1III). In a prototype cell configuration with an NCM811 cathode and a thin Li foil anode, the SSNE demonstrates exceptional performance: it maintains 81.4% capacity retention over 200 cycles at room temperature, exhibits rate capability enduring up to 2C, and delivers stable operation for 190 cycles in a 1.2 Ah pouch cell. Incorporating dynamic polyrotaxane chemistry into a supramolecular electrolyte framework establishes a novel molecular‐to‐system strategy that optimizes ion transport, enforces spatial confinement, and enhances interfacial stability across multiple scales for next‐generation high‐voltage SLMBs.

## 2 Results and Discussion

### 2.1 Design and Characterizations of Polyrotaxane (PR)‐Functionalized SSNE

The structural design of the TPU‐rotaxane is illustrated in Figure S1. The preparation of the SSNE involves the sequential polymerization of PR and the formation of a semi‐interpenetrating network, as shown in Figure S1 and Scheme S3. The synthesis of the PR consists of two primary stages: (1) the formation of a pseudo‐polyrotaxane via threading of the macrocyclic 18C6 unit onto polytetrahydrofuran (PTHF) chains driven by hydrogen bonding interactions between the oxygen atoms of 18C6 and the terminal ─OH groups of PTHF; (2) catalyzed by dibutyltin dilaurate, the terminal hydroxyl groups of the pseudo‐polyrotaxane undergo reaction with isocyanate groups (─N═C═O), thereby effecting end‐capping and initiating the subsequent polymerization of thermoplastic polyurethane (TPU) to yield PR structure. The polymer chain was subsequently extended using hydroxyborate (Scheme S1) and capped with BTFB to enhance structural stability. The product exhibits good solubility and physicochemical stability. After purification, the PR was mixed with DOL, 2,4,6‐tris(oxiran‐2‐ylmethoxy)‐1,3,5‐triazine (TTE, Scheme S2) crosslinker, and Sc(OTF)_3_ as an initiator to form a stable precursor solution. LiTFSI was then added, and the mixture was stirred to obtain a homogeneous electrolyte solution. This solution was assembled into a CR2016 cell case and subjected to in situ polymerization at 60°C. Finally, the electrolyte was cured in an oven to ensure uniformity and stability for electrochemical performance testing. To precisely characterize the structure of TPU‐rotaxane, a reference sample (TPU‐model) was prepared, in which the only difference from TPU‐rotaxane lies in the sequence of 18C6 incorporation, while all other synthetic steps remain identical (Scheme S4). Comparative characterization results for TPU‐rotaxane and TPU‐model validated the structural features of the target product.

**FIGURE 1:**
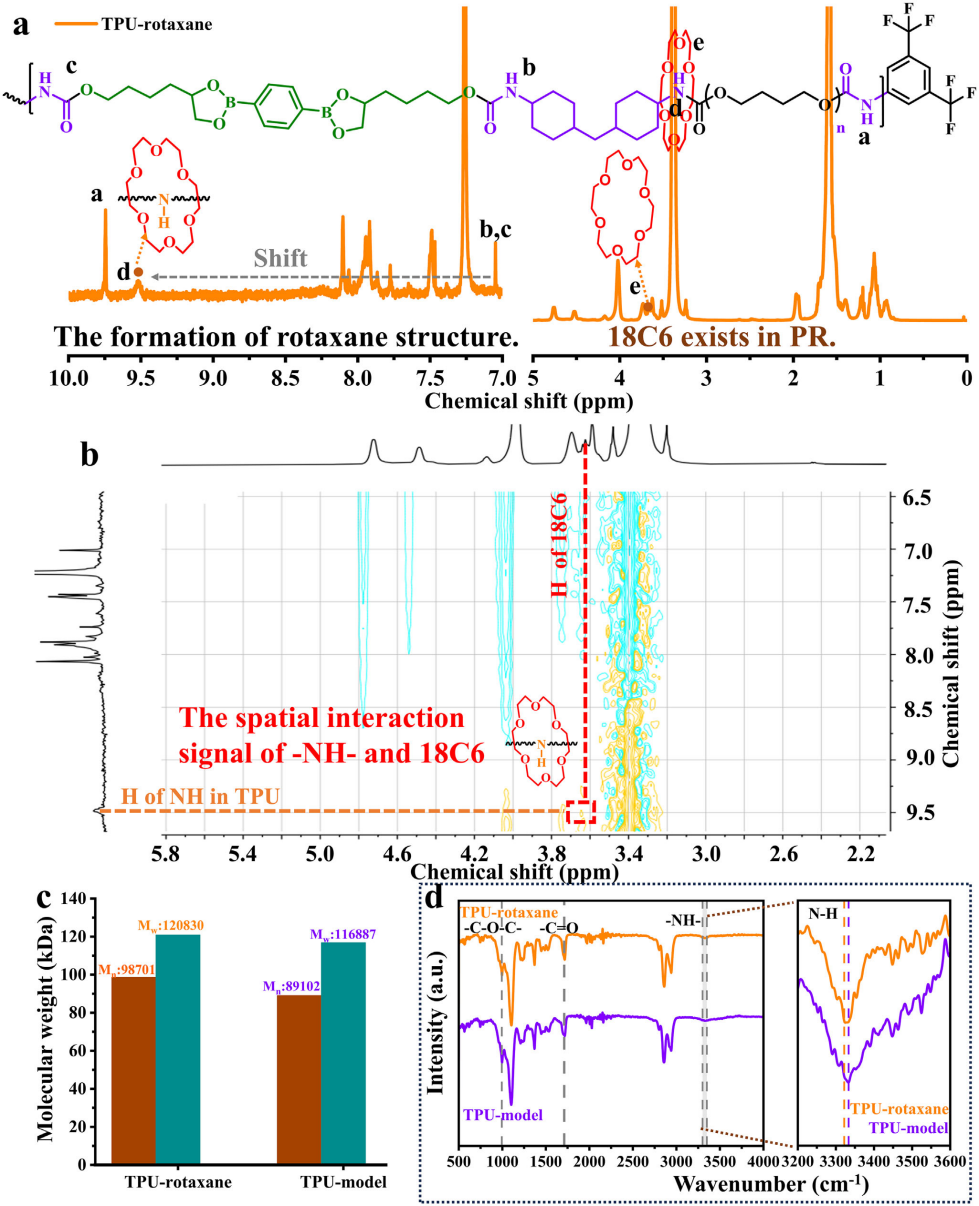
The structural characterization of TPU‐rotaxane. (a) ^1^H NMR spectrum of TPU‐rotaxane. (500 MHz, CDCl_3_, R.T.) (b) NOESY spectrum of TPU‐rotaxane. (c) The molecular weights of TPU‐rotaxane and TPU‐model. (d) FT–IR spectra of TPU‐rotaxane and TPU‐model. (The right panel shows a magnified region (×10) displaying FT–IR peaks associated with the NH bonds of the samples).

As shown in Figure 1a, ^1^H NMR analysis of TPU‐rotaxane reveals the presence of ‐NH‐ groups formed from the reaction of isocyanate with PTHF and hydroxyborate (chemical shift at 7.04 ppm), as well as with the capping agent BTFB (chemical shift at 9.74 ppm). Additionally, the proton signals of 18C6 (3.66 ppm) and certain ─NH─ groups exhibited a downfield shift (from 7.04 to 9.51 ppm), which can be attributed to hydrogen‐bonding‐induced deshielding within 18C6. In sharp contrast, no such shift was observed in the ^1^H NMR spectrum of TPU‐model (Figure S11), further validating the formation of the PR structure. Moreover, NOESY analysis (Figure 1b) revealed distinct spatial correlations between the proton signals of 18C6 and ─NH─ groups of TPU (indicated by the red dashed rectangle), providing direct evidence of the PR structure. No such correlations were observed in the NOESY spectrum of the TPU‐model (Figure S12). Size exclusion chromatography (SEC) was used to analyze the molecular weights of the two polymers (Figure 1c; Figure S13). TPU‐rotaxane exhibited a slightly higher molecular weight than that of TPU‐model, due to the incorporation of 18C6 on the polymer backbone, which increased hydrodynamic volume and, consequently, the apparent molecular weight. FTIR analysis (Figure 1d) revealed a red shift of the secondary amine absorption peak near 3300 cm^−1^ for TPU‐rotaxane as compared to TPU‐model [48, 49]. This red shift indicates the presence of a hydrogen‐bond‐stabilized rotaxane structure, consistent with ^1^H NMR and NOESY results. Differential scanning calorimetry (DSC, Figure S14) revealed that TPU‐rotaxane exhibited a slightly lower glass transition temperature (T_g_) compared to TPU‐model. This decrease in T_g_ is attributed to the rotaxane structure weakening the non‐covalent interactions between TPU chains, thereby lowering the energy barrier for segmental motion and reducing T_g_.

### 2.2 Ionic Diffusion and Mechanical Behaviors of SSNE

**FIGURE 2:**
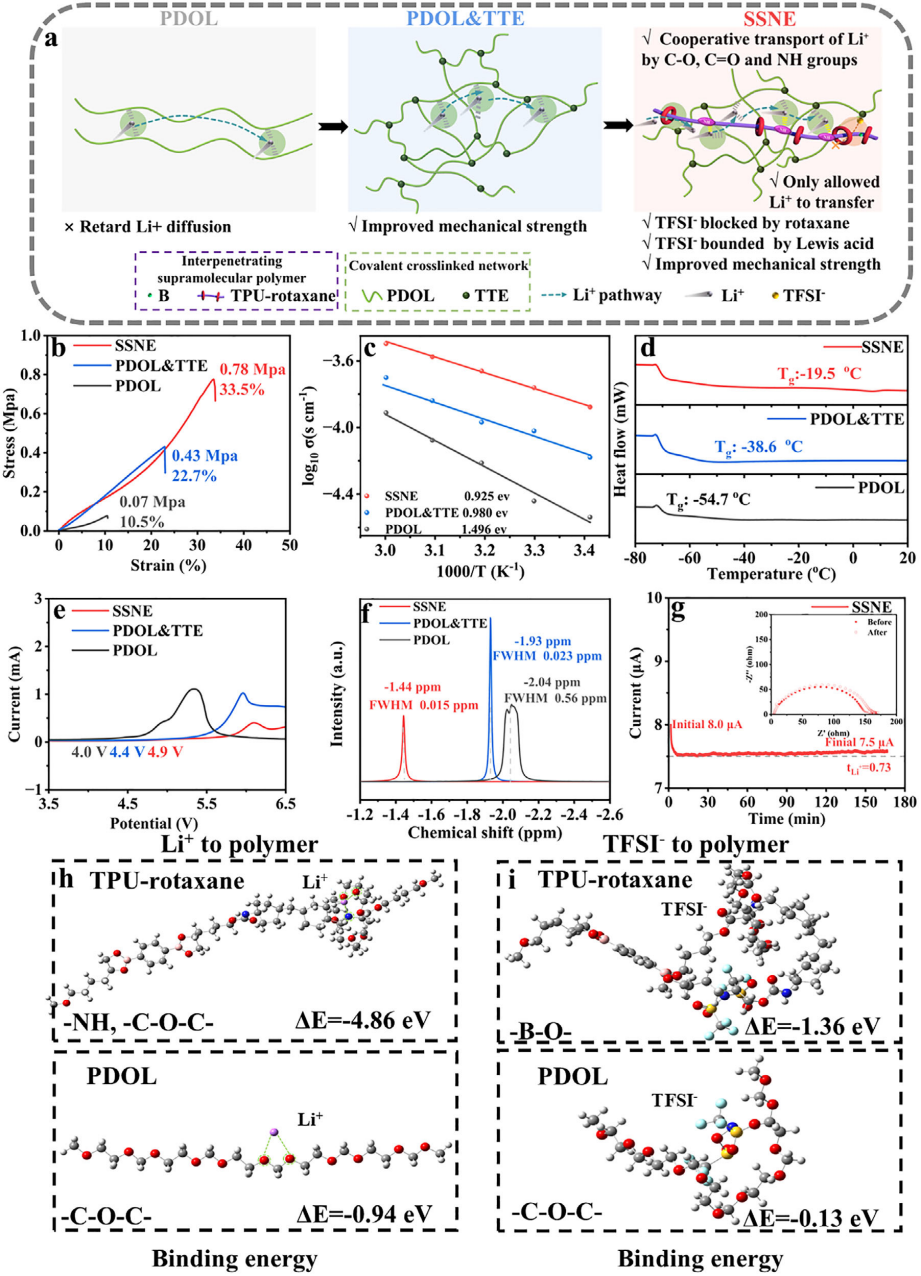
Characterization of the physicochemical properties of SPEs. (a) Schematic illustrations of the Li^+^ conduction mechanisms of linear PDOL, crosslinked PDOL & TTE, and semi‐interpenetration SSNE. (b) Stress‐strain curves of SPEs. (c) Ionic conductivities of SPEs fitted by the Arrhenius equation. (d) DSC curves of SPEs. (e) LSV curves of SPEs. (f) ^7^Li NMR spectra of the Li^+^ chemical environment in three precursor solutions before polymerization. (g) Li^+^ transference number of SSNE electrolyte measured by the potential static polarization method (inset images: EIS of the symmetrical cell before and after polarization). (h) The binding energies and corresponding adsorption configurations of Li^+^ to TPU‐rotaxane and PDOL chains. (i) The binding energies and corresponding adsorption configurations of TFSI^−^ to TPU‐rotaxane and PDOL chains.

Figure 2a illustrates the enhancement of PDOL performance through crosslinking and functionalization in TPU‐rotaxane. Compared with the PDOL‐based system, where Li^+^ transport primarily relies on coordination and dissociation with ether oxygen groups, the PR structure in TPU‐rotaxane, together with its conductive functional groups (─C═O and ─NH─), introduces additional Li^+^ transport pathways beyond ether oxygen coordination. These supplementary coordination sites facilitate LiTFSI dissociation and synergistically promote ion diffusion. Additionally, the borate ester part in TPU effectively immobilizes TFSI^−^, further improving conductivity. Compared to PDOL, the SSNE electrolyte exhibits significantly higher mechanical strength (0.78 MPa) and toughness (33.5% fracture elongation) due to the crosslinking of PDOL and its semi‐interpenetrating network. These values represent substantial enhancements compared with those of PDOL (0.07 MPa, 10.5%) (Figure 2b). Figure 2c shows the ionic conductivities and Arrhenius fits for the SPEs. The ionic conductivity of SSNE reaches 0.18 mS cm^−1^ at 25°C, nearly an order of magnitude higher than that of pure PDOL (0.03 mS cm^−1^). Moreover, SSNE exhibits the lowest activation energy (E_a_ = 0.925 eV) among the SPEs, indicating accelerated Li^+^ diffusion kinetics. ESI tests conducted between 20 and 60°C (Figure S15) confirmed this trend. DSC analysis (Figure 2d) shows that crosslinking, along with high T_g_ of TPU‐rotaxane components, increases the rigidity of SSNE, resulting in a T_g_ of −19.5°C, which is higher than that of PDOL & TTE and PDOL. Despite the decreased segmental mobility, multiple conduction mechanisms enable SSNE to maintain high room‐temperature ionic conductivity, compared to PDOL.

LSV curves (Figure 2e) revealed that the electrochemical stability windows of SSNE, PDOL & TTE, and PDOL were 4.9, 4.4, and 4.0 V, respectively. The enhanced stability of PDOL & TTE compared to PDOL is likely due to the high‐voltage tolerance of TTE, which suppresses polyether backbone decomposition at the cathode. ^7^Li NMR spectra reveal that the full width at half maximum of SSNE (0.015 ppm) was significantly narrower than those of PDOL & TTE (0.023 ppm) and PDOL (0.560 ppm), indicating that the SSNE reduced the negative charge density around Li^+^ (Figure 2f). The peak shift of SSNE to a low field, −1.44 ppm (compared to PDOL & TTE at −1.93 ppm and PDOL at −2.04 ppm), confirms weaker electrostatic interactions between TFSI^−^ and Li^+^, which benefits lithium ion transport. Figure 2g and Figures S16 and S17 demonstrate that SSNE exhibits a much higher $t_{Li^{+}}$ (0.73) than PDOL & TTE (0.49) and PDOL (0.46). This improvement is attributed to the restricted mobility of TFSI^−^ in SSNE. The rotaxane structure spatially confines TFSI^−^ anions, as their radius (3.30 Å) is significantly larger than the cavity of the 18C6 host molecule (1.60 Å), preventing their entry and movement. In contrast, smaller Li^+^ ions (radius 0.76 Å) can selectively traverse the structure [50, 51, 52]. Regulation of the local electron density in the electrolyte can weaken the Li^+^‐TFSI^−^ interaction and optimize the coordination environment of Li^+^. In TPU‐rotaxane, strong electron‐withdrawing sites (such as boron Lewis acid sites) effectively reduce the electrostatic binding between Li^+^ and TFSI^−^ anions, creating a more favorable local “electric field environment” for Li^+^ transport. This electronic modulation promotes lithium salt dissociation, enabling more Li^+^ to migrate in a free state rather than cooperatively with anions, thereby significantly enhancing the lithium‐ion transference number [53, 54]. Density functional theory (DFT) calculations (Figure 2h,i) revealed that Li^+^ binds more strongly to TPU‐rotaxane (−4.86 eV) than to PDOL (−0.94 eV). Moreover, TFSI^−^ adsorption on the ─O─B─O─ group of TPU‐rotaxane (−1.36 eV) is significantly stronger than on the C‐O‐C group of PDOL (−0.13 eV). These results further support that TFSI^−^ mobility is restricted in SSNE, thereby facilitating Li^+^ migration.

### 2.3 Cathode/Li Foil‐ SSNE Electrolyte Interfacial Stabilities across the Scale

**FIGURE 3:**
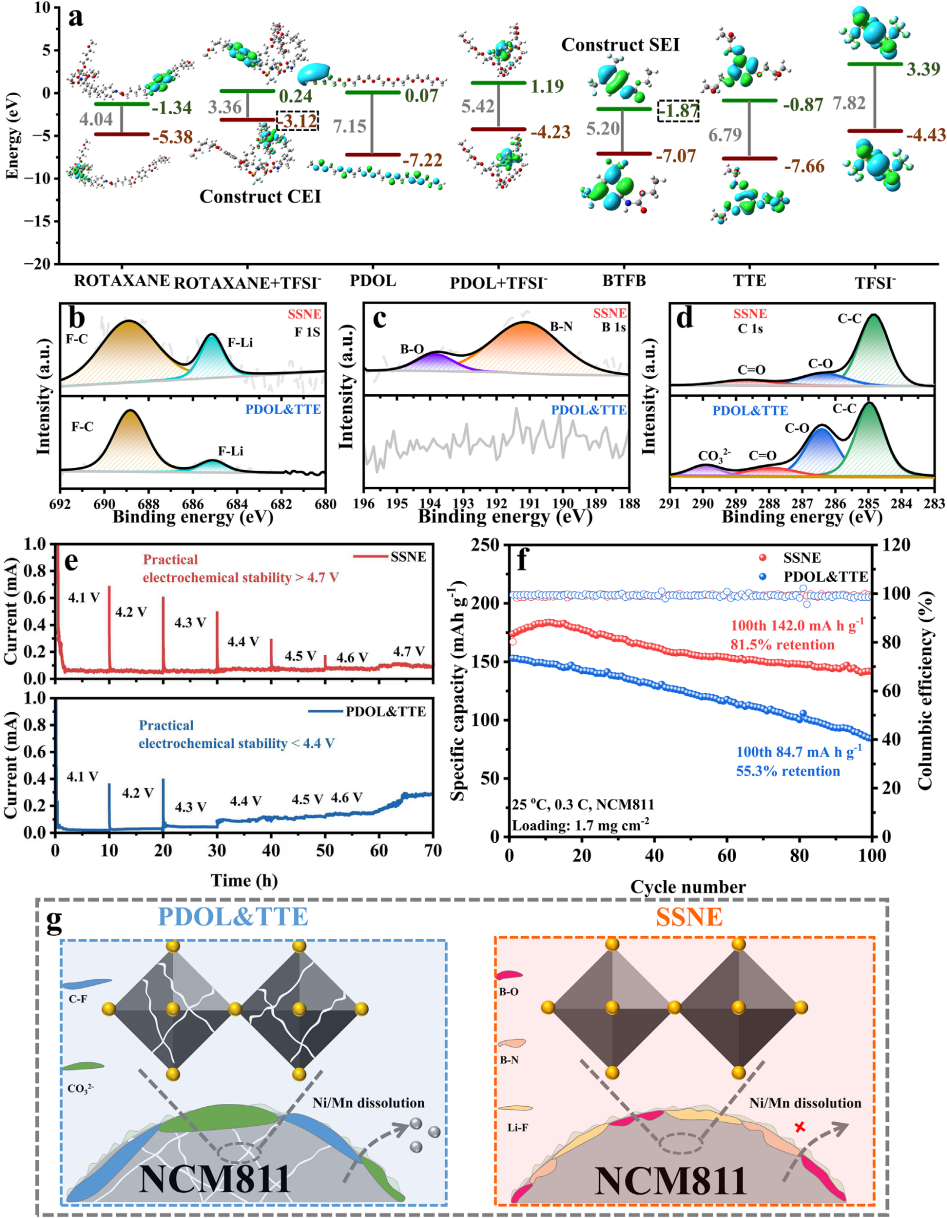
(a) Theoretical calculations of LUMO/HOMO values and corresponding geometry structures of the SPEs components. (b–d) XPS analysis of the cycled NCM811 surface, showing (b) F 1s, (c) B 1s, and (d) C 1s Spectra. (e) Floating tests of SSNE and PDOL & TTE. (f) Cycling performances of NCM811|SSNE|Li and NCM811|PDOL&TTE|Li cells within the voltage range of 2.8–4.5 V. (g) Schematic illustration showing the structural evolution of the cycled NCM811.

The electrochemical stability of SSNE was systematically evaluated by analyzing electrolyte components. DFT calculations were performed to determine the molecular orbital energy levels of TPU‐rotaxane (ROTAXANE), its TFSI^−^ complex (ROTAXANE & TFSI^−^), PDOL, PDOL & TFSI^−^, BTFB, TTE, and TFSI^−^ (Figure 3a). The highest occupied molecular orbital (HOMO) and lowest unoccupied molecular orbital (LUMO) energy levels serve as key descriptors of the redox behavior. The component possessing the highest HOMO is preferentially oxidized at the cathode, thereby generating a stable CEI that suppresses polymer decomposition and inhibits metal dissolution. Besides, the component with the lowest LUMO is preferentially reduced at the anode, thereby generating a uniform SEI that facilitates Li^+^ diffusion, suppresses dendrite formation, and enhances cycling stability. DFT calculations revealed that the HOMO energy levels of ROTAXANE, ROTAXANE & TFSI^−^, PDOL, PDOL & TFSI^−^, BTFB, TTE, and TFSI^−^ were −5.38, −3.12, −7.22, −4.23, −7.07, −7.66, and −4.43 eV, respectively. These values suggest that in SSNE, ROTAXANE & TFSI^−^ are preferentially oxidized to form the CEI. The LUMO levels are −1.34, 0.24, 0.07, 1.19, −1.87, −0.87, and 3.39 eV, respectively, suggesting that in SSNE, BTFB is preferentially reduced to form the SEI. Moreover, the electrochemical stability window of the PDOL & TTE system is significantly higher than that of PDOL due to the introduction of TTE. TTE possesses a lower HOMO energy level, indicating a reduced tendency toward electron loss and oxidative decomposition under high potentials. When TTE forms a eutectic system with PDOL, electron extraction from the electrolyte becomes more difficult at high cathode voltages. Consequently, the intrinsic electronic structure of the electrolyte is stabilized, leading to a markedly enhanced high‐voltage tolerance of the PDOL & TTE system compared with PDOL [55, 56]. The composition of CEI was further analyzed by X‐ray photoelectron spectroscopy (XPS). The PDOL & TTE system exhibited a higher content of organic C‐F species at 688.5 eV, indicating ‐CF_3_ in TFSI^−^. In contrast, the SSNE system exhibited a high concentration of inorganic Li─F species at 684.8 eV, suggesting that SSNE promotes the formation of a stable, LiF‐rich CEI, effectively protecting the cathodes from degradation (Figure 3b). XPS B 1s spectra detected B─N and B─O bonds on the SSNE cathode surface, confirming the formation of a B‐containing CEI; no such signals were observed in the PDOL & TTE system (Figure 3c). C 1s spectra exhibited peaks at 284.8, 286.4, 288.2, and 290.6 eV, corresponding to C─C, C─O─C, ─COO, and Li_2_CO_3_ species (Figure 3d). Strong signals at 290.6 eV (Li_2_CO_3_) and 288.2 eV (‐COO) indicated that PDOL decomposition products dominated the cathode surface chemistry in the PDOL & TTE system. In contrast, the intensity of these carbonate‐related peaks was substantially reduced in the SSNE system, demonstrating that PDOL decomposition was effectively suppressed. The stability of the electrolyte‐cathode interface was further assessed through electrochemical floating analysis (Figure 3e). For the NCM811|PDOL & TTE|Li cell, a sharp increase in the leakage current was observed beyond 4.4 V, indicating interfacial side reactions. By comparison, the NCM811|SSNE|Li cell maintained a stable leakage current up to 4.7 V, suggesting that SSNE can effectively suppress high‐voltage interfacial degradation. In the voltage range of 2.8–4.5 V (Figure 3f), the NCM811|SSNE|Li cell delivered a reversible capacity of 142.0 mAh g^−1^ over 100 cycles at room temperature, with 81.5% capacity retention. This performance is markedly superior to that of the NCM811|PDOL & TTE|Li cell, which retained only 55.3% of its initial capacity under the same conditions due to its poor high‐voltage stability. The cathodes of SSNE‐ and PDOL & TTE‐based cells were further examined after cycling. As shown in Figure S18a,b, the CEI formed in SSNE was thinner (9 nm) and more electrochemically stable than that formed in PDOL & TTE (30 nm). High‐resolution transmission electron microscope (HRTEM) analysis (Figure S18c) revealed that the SSNE‐based cathode retained an intact lattice structure after cycling, whereas the PDOL & TTE‐based cathode showed significant structural damage after cycling (Figure S18d). These HRTEM observations were corroborated by selected‐area electron diffraction (SAED) patterns. SSNE‐based cathodes exhibited clear single‐crystal diffraction spots (Figure S18e), indicating preserved crystallinity, while the PDOL & TTE sample displayed a diffuse polycrystalline ring instead of sharp spots (Figure S18f), signifying severe structural degradation. X‐ray diffraction (XRD) analysis (Figure S19) supported these findings. After 100 cycles at 4.5 V, the SSNE cathode maintained its original crystal structure, closely matching the diffraction pattern of pristine NCM811. In contrast, the PDOL & TTE cathode showed an attenuated and shifted (003) peak, indicating significant structural deterioration. Electrolyte coupling with an NCM811 cathode leads to issues such as unstable high‐voltage electrode/electrolyte interfaces, Li^+^/Ni^2+^ cation mixing, severe intergranular microcracking, and dissolution of transition‐metal cations. The poor oxidative stability of SPEs such as PDOL fails to address these issues, thereby limiting their electrochemical performance (Figure 3g). However, SSNE mitigates these issues by in situ forming a robust and ultrathin cathode–electrolyte interphase (CEI) enabled by the rational design of TPU‐rotaxane.

**FIGURE 4:**
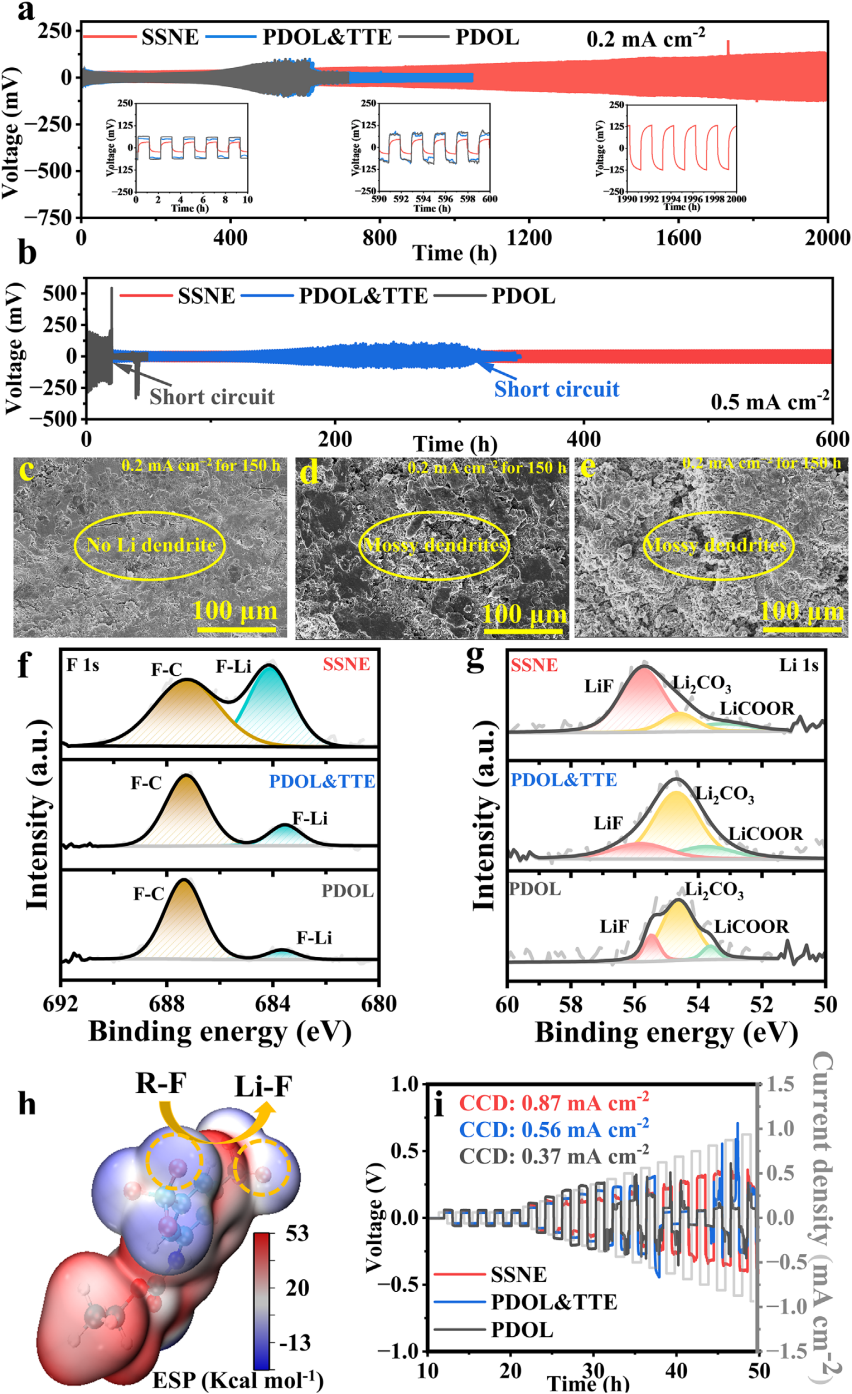
Interfacial electrochemical stability between the SPEs and Li anodes. (a) Voltage profiles of the symmetric cells with different SPEs at 0.2 mA cm^−2^. (b) Voltage profiles of the Li symmetric cells with different SPEs at 0.5 mA cm^−2^. (c–e) Top‐view SEM images of Li foil disassembled from Li|SPEs|Li, after symmetric cycling at 0.2 mA cm^−2^ for 150 h, (c) SSNE cell, (d) PDOL & TTE cell, (e) PDOL cell. (f) F 1s, spectra of Li foil disassembled from the Li|SPEs|Li cells after operation for 100 h. (g) Li 1s spectra of Li foil disassembled from the Li|SPEs|Li cells after running for 100 h. (h) Electrostatic potential (ESP) distribution of BTFB. (i) Galvanostatic Li plating/stripping profiles of Li symmetric cells with SPEs at step‐increased current densities.

The SSNE Li||Li symmetric cell exhibited outstanding cycling stability at room temperature, maintaining stable operation for over 2000 h at a current density of 0.2 mA cm^−2^ with a polarization voltage of approximately 35 mV. In contrast, cells employing PDOL & TTE and PDOL electrolytes failed after 660 and 603 h, respectively, accompanied by pronounced voltage fluctuations. When the current density was increased to 0.5 mA cm^−2^ (Figure 4b), the SSNE Li||Li symmetric cell continued to deliver a stable polarization voltage (∼47 mV) for more than 600 h, whereas the PDOL & TTE‐ and PDOL‐based cells failed after 300 and 30 h, respectively, with significantly elevated polarization voltages. Post‐mortem morphological analyses after 150 h of Li plating/stripping (Figure 4c–e) revealed a smooth and compact surface in the SSNE cell, while the PDOL & TTE and PDOL counterparts displayed evident mossy and dendritic Li growth. Subsequent surface compositional analyses further elucidated the chemistry of the SEI. The F 1s spectra of the Li||Li symmetric cells exhibited characteristic peaks at 688.5 and 684.8 eV, corresponding to ‐CF_3_ in LiTFSI and LiF (Figure 4f). The Li 1s spectra showed three distinct peaks at 55.9, 55.2, and 54.2 eV, attributable to LiF, Li_2_CO_3_, and LiCO_2_R species, respectively (Figure 4g). These findings indicate that the in situ‐formed SEI is composed of polyether‐based organic moieties embedded with rigid inorganic LiF domains. HRTEM (Figure S20) confirmed the coexistence of C and F elements, verifying the formation of a LiF‐rich SEI. Figure S21 demonstrated a homogeneous distribution of LiF nanocrystals on the Li surface after cycling, which effectively lowers the Li^+^ diffusion barrier and ensures uniform ionic transport. ESP analysis of the BTFB molecule (Figure 4h) revealed negative potential concentration around F atoms, making F─C bonds prone to cleavage and promoting LiF formation in the SEI. Finally, limiting‐current measurements of the three SPEs (Figure 4i) showed that the SSNE cell achieved a critical current density of 0.87 mA cm^−2^, outperforming those of PDOL & TTE (0.56 mA cm^−2^) and PDOL (0.37 mA cm^−2^), due to its superior interfacial stability and ionic transport capability.

### 2.4 Electrochemical Performance of High‐Voltage Lithium Batteries

**FIGURE 5:**
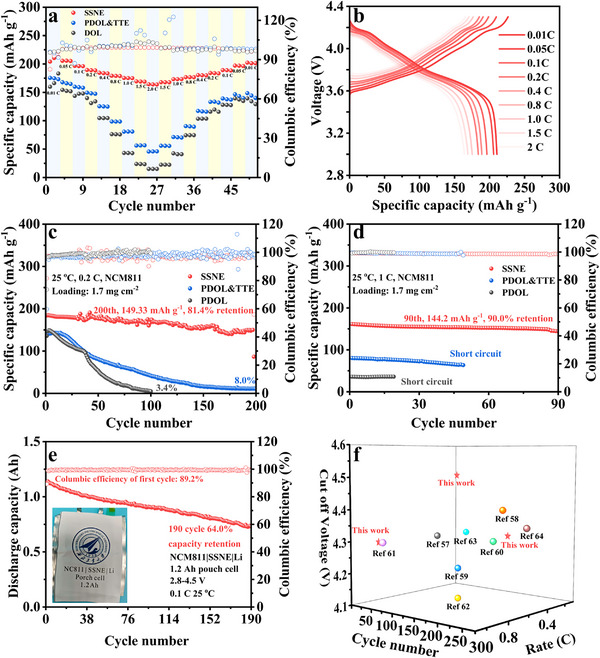
Electrochemical performance of SPEs. (a) Rate performance of NCM811||Li cell with SSNE, PDOL & TTE and PDOL. (b) The charge and discharge curves of SSNE at different rates. (c, d) Cycling performance of NCM811||Li full cell with SSNE PDOL & TTE and PDOL electrolytes rates of (c) 0.2C and (d) 1.0C. (e) Cycling performance of the NCM811|SSNE|Li pouch cell within the voltage range of 2.8–4.5 V. (f) Comparison of the cut‐off voltage, rate, and cycle number of NCM811|SSNE|Li cells with previous reported examples.

Benefiting from its high voltage tolerance and facile interfacial kinetics toward the Li foil, the NCM811||Li cell employing SSNE electrolyte exhibited exceptional rate performance (Figure 5a,b), delivering stable capacities across various rates. At a cut‐off voltage of 4.3 V, the SSNE‐based achieved discharge capacities of 189.4 mAh g^−1^ at 0.2C, 175.3 mAh g^−1^ at 1.0C, and 163.8 mAh g^−1^ at 2.0C, respectively, significantly surpassing those of NCM811|PDOL & TTE|Li and NCM811|PDOL|Li. When the rate was returned to 0.1C, the SSNE cell recovered a specific capacity of 188.5 mAh g^−1^, whereas the PDOL‐based cell regained only 128.0 mAh g^−1^, corroborating the superior structural and interfacial stability of the SSNE electrolyte. At room temperature and a rate of 0.2C (Figure 5c; Figure S22), the NCM811 full cells using PDOL & TTE and PDOL electrolytes exhibited rapid capacity fading, retaining merely 8.0% and 3.4% of their initial capacities, respectively. In contrast, the SSNE‐based cell preserved 81.4% of its initial capacity after 200 cycles. Even under higher‐rate conditions of 1C (Figure 5d; Figure S23), the SSNE cell delivered a higher initial capacity (161.0 mAh g^−1^), approximately double that of PDOL & TTE (80.4 mAh g^−1^) and PDOL (65.7 mAh g^−1^), and maintained 90% of its capacity over 90 stable cycles. To further validate its practical applicability, a laminated pouch cell was fabricated using an NCM811 cathode (3.7 mAh cm^−2^), a 20 µm PE separator impregnated with SSNE electrolyte, and a thin Li anode (25 µm, 4.9 mAh cm^−2^) with an anode/cathode (N/P) capacity ratio of 1.3 (Table S1). The pouch cell exhibited acceptable cycling stability at room temperature, operating for 190 cycles without experiencing short circuits. (Figure 5e). Moreover, when paired with a lithium iron phosphate (LFP) cathode, the SSNE electrolyte enabled superior cycling and rate performances across multiple current densities, outperforming both PDOL & TTE and PDOL systems (Figures S24–S27). Compared with previously reported ether‐based NCM811||Li cells (Figure 5f), the NCM811|SSNE|Li cell exhibited superior cycling performance, even under harsh conditions, particularly at high current densities or elevated cut‐off voltages [57, 58, 59, 60, 61, 62, 63, 64]. The safety of the SSNE‐based pouch cell was further confirmed under extreme conditions (Figure S28). After charging to 4.3 V, the NCM811|SSNE|Li pouch battery light a small bulb and maintained reliable operation and stable voltage even when bent or folded.

## 3 Conclusion

In conclusion, a supramolecular SSNE was developed to overcome the intrinsic limitations of conventional polyether‐based SPEs by integrating rotaxane‐functionalized polyurethane chains within a crosslinked PDOL framework. This hierarchical architecture enables multiscale Li^+^ transport through the concerted action of dynamic host‐guest interactions and Lewis acid coordination, ensuring both high mobility and structural stability. As a result, the SSNE exhibits an exceptional ionic conductivity of 0.18 mS cm^−1^, a Li^+^ transference number of 0.73, and mechanical robustness of 0.78 MPa at 50 µm thickness. The TPU‐rotaxane/TFSI^−^ complexes effectively stabilize the CEI up to 4.9 V, while the BTFB part promotes the formation of a highly diffusive and resilient SEI layer on the Li anode. When assembled in NCM811|SSNE|Li cells, the system delivers stable cycling with 81.4% capacity retention over 200 cycles, rate capability up to 2C, and 1.2 Ah pouch‐cell performance maintained over 190 cycles at room temperature. Collectively, this work bridges supramolecular chemistry and macroscopic electrochemical functionality, offering a generalizable design strategy for next‐generation high‐voltage SLMBs.

## Conflicts of Interest

The authors declare no conflicts of interest.

## Supporting information




**Supporting File**: advs74374‐sup‐0001‐SuppMat.docx.

## Data Availability

The data that support the findings of this study are available from the corresponding author upon reasonable request.
