# Supplementary material for: Polyrotaxane‐Assembled Semi‐Interpenetrating Polymer Electrolytes Enabling High‐Voltage Lithium Metal Batteries
Source: Adv Sci (Weinh). 2026 Feb 26;13(24):e21892. doi: 10.1002/advs.202521892 (PMC13116361; doi:10.1002/advs.202521892)
Supplement: Supplementary file 1 — Supporting File: advs74374‐sup‐0001‐SuppMat.docx. [file ADVS-13-e21892-s001.docx]

**Supporting Information**

**Polyrotaxane-Assembled Semi-Interpenetrating Polymer Electrolytes Enabling High-Voltage Lithium Metal Batteries**

*Tianyi Wang^a^*, *Yue Ma^b*^*, *Jiacheng Liu^b^*, *Zhaofan He^a^*, *Jin Liang^a^*, *Kailiang Ren^a^*, *and Jie Kong^a*^*

^a^Shaanxi Key Laboratory of Macromolecular Science and Technology, School of Chemistry and Chemical Engineering, Northwestern Polytechnical University, Xi’an 710072, P. R. China.

^b^School of Environmental and Chemical Engineering, Xi‘an Polytechnic University, Xi'an, 710048 China

*Corresponding Authors, E-mail: mayue04@nwpu.edu.cn (Y.M.), kongjie@nwpu.edu.cn (J.K.)

1. General information of materials

Polytetrahydrofuran (PTHF, Mn=2,000 Da), bis(trifluoromethanesulfone)imide (LiTFSI), 1-methyl-2-pyrrolidinone (NMP), poly(vinylidene fluoride) (PVDF), super C65 carbon, dicyclohexylmethane 4,4'-Diisocyanate (DDI, mixture of isomers), 1,4-benzenediboronic acid, hexane-1,2,6-triol, cyanuric chloride, glycidol, sodium hydride (NaH in mineral oil 60% purity), tetrahydrofuran (THF, water ≤ 50 ppm), 1,3-dioxolane (DOL, water ≤ 50 ppm), 1-isocyanato-3,5-bis(trifluoromethyl)benzene (BTFB), dibutyltin dilaurate (DBTDL), 1,4,7,10,13,16-hexaoxacyclooctadecane (18C6), scandium trifluoromethanesulfonate (Sc(OTF)_3_) were purchased from Aladdin, Macklin or Innochem. All reagents were used without further purification. Lithium metal was purchased from China Energy Lithium with a thickness of 50 or 25 μm. All reactions were carried out under the Schlenk technique.

2. Synthetic procedures

**2.1 The structure design concept of TPU-rotaxane**

**Figure S1.** Schematic diagram of the structure design of TPU-rotaxane.

**2.2 Schematic diagram of the preparation process of the SSNE electrolyte cell**

**Figure S2.** Schematic diagram of the preparation process of the SSNE electrolyte battery.

**2.3 Synthesis of** **2,2’-(1,4-phenylene)-bis[4-(4-hydroxybutyl)-1,3,2-dioxaborolane (HDB)**

**Scheme S1.** Synthetic route of HDB.

The synthesis steps of HDB as the same as Song et al.^[S1]^ The structure is confirmed by the NMR data. ^1^H ^13^C and ^11^B spectra data. ^1^H NMR (CDCl_3_, 500 MHz), δ 7.72 (s, 1H), 4.46 (qd, J = 7.2, 4.9 Hz, 1H), 4.31 (t, J = 8.4 Hz, 1H), 3.83 (dd, J = 9.0, 7.2 Hz, 1H), 3.53 (t, J = 6.3 Hz, 1H), δ 1.63 (dq, J = 13.3, 6.8, 6.0 Hz, 2H), 1.58 – 1.39 (m, 2H), 1.39 – 1.29 (m, 2H). ^11^B NMR (CDCl_3_, 160 MHz), δ 26.67. ^13^C NMR (CDCl_3_, 126 MHz), δ 134.01, 77.52, 71.12, 62.27, 35.80, 32.35, 21.33.

**2.4 Synthesis of 2,4,6-tris(oxiran-2-ylmethoxy)-1,3,5-triazine (TTE)**

**Scheme S2.** Synthetic route of TTE.

The TTE was prepared by the Williamson synthesis of cyanuric chloride with glycidol. A solution of NaH (in mineral oil, 60% purity, 160 mg, 4 mmol) in THF (5 mL) was added dropwise to a solution of glycidol (260 mg, 3.5 mmol), in THF (5 mL) at 0 °C. After complete addition, the reaction mixture was allowed to stir for 6 h at room temperature. Then cyanuric chloride (288 mg, 1mmol) was added to the solution and stirred for a further 3 h. When bubble evolution ceased, the temperature was raised to reflux conditions and maintained for 12 h. After cooling to room temperature, the salt formed during the reaction was removed by suction filtration, and the product was subsequently isolated and purified by column chromatography (150 mg, 0.5 mmol, yield 50% ). The product was characterized by ^1^H NMR, ^13^C NMR and HRMS spectroscopies. ^1^H NMR (CDCl_3_, 500 MHz), δ 4.59 (dd, J = 12.0, 3.3 Hz, 1H), 4.26 (dd, J = 12.0, 6.1 Hz, 1H), 3.30 (dq, J = 6.5, 3.2 Hz, 1H), 2.82 (t, J = 4.5 Hz, 1H), 2.66 (dd, J = 4.9, 2.6 Hz, 1H). ^13^C NMR (CDCl_3_, 126 MHz) δ 172.85, 69.01, 49.01, 44.64. HRMS: calculated 297.09, found [M+H]^+^ 298.10.

**2.5 Synthesis of rotaxane polyurethane (TPU-rotaxane)**

**Scheme S3.** Synthetic route of TPU-rotaxane.

TPU-rotaxane was synthesized via the condensation of PTHF with DDI. Briefly, 2.5 g (1.25 mmol) of PTHF and 2.5 g (9.46 mmol) of 18C6 were added into a 100 mL Schlenk tube and stirred at 90 °C for 3 h to form a pseudorotaxane. Then cooled to 60 ^o^C, 524 mg (2.00 mmol) of DDI was added, and 5 μL of DBTDL was added to the solution as a reaction catalyst. THF was added to adjust the viscosity of the solution. After stirring for 4 hours, HDB 400mg (0.9 mmol) was added as a chain extender, followed by the addition of 5 μL of DBTDL as a catalyst, and continued stirring for another 4 hours. Subsequently, add BTFB as an end-capping agent. After cooling to room temperature, THF was removed by rotary evaporation, and purified by dialyzed against ethanol for 48 h using a dialysis membrane with a molecular weight cutoff (MWCO) of 2,000 Da. The polymer structure was characterized using ^1^H NMR, SEC, and NOESY tests.

**2.6 Synthesis of model polymer polyurethane (TPU-model)**

**Scheme S4.** Synthetic route of TPU-model.

The formation of the TPU-model was similar to the procedure of synthesizing TPU-rotaxane. In brief: 2.5 g (1.25 mmol) of PTHF, 524 mg (2.00 mmol) of DDI and 5 μL of DBTDL were added to a 100 mL Schlenk tube and stirred at 60 ^o^C for 4h THF was added to adjust the viscosity of the solution. Then, HDB 400 mg (0.9 mmol) was added as a chain extender, followed by the addition of 5 μL of DBTDL as a catalyst and continued stirring for another 4 h. Subsequently, BTFB was added as an end-capping agent. Then the temperature was raised to 90 ^o^C, 2.5 g (9.46 mmol) of 18C6 were added and stirred for another 3h. After that, the solution was diluted with 20 mL of ethanol and dialyzed for 48 h with a dialysis membrane (MWCO of 2,000 g mol^-1^) by using ethanol, and used for ^1^H NMR, NOESY, and SEC tests.

**2.7 Preparation of polymer electrolyte** **precursor solution**

SSNE precursor solution was prepared by dissolving 600 mg TPU-rotaxane, 20 mg TTE, 30 mg Sc(OTF)_3,_ and 670 mg LiTFSI in 2 mL DOL, vigorously stirred for 4 h at room temperature, to form a homogeneous solution. PDOL & TTE precursor solution was prepared by dissolving 20 mg TTE, 30 mg Sc(OTF)_3,_ and 520 mg LiTFSI in 2 mL DOL. The preparation of the PDOL precursor solution is identical to that of PDOL & TTE, with the exception of omitting the crosslinking agent TTE. The salt concentration for both SSNE, PDOL & TTE, and PDOL was kept constant in all the cases at a molar ratio of 15:1 [-CH_2_CH_2_O- (EO) ]/[Li^+^]].

**Figure S3**: Room-temperature ionic conductivity of the electrolyte at different EO/Li ratios.

Based on the literature survey, the optimal EO/Li ratio for polyether-based electrolytes generally falls within the range of 20:1 to 10:1, while either a higher or lower EO/Li ratio results in reduced ionic conductivity. ^[S2-S4]^ Therefore, in this work, TPU-rotaxane was blended with DOL, followed by the addition of the corresponding lithium salt, and EO/Li ratios of 10:1, 15:1, and 20:1 were systematically investigated. The results indicate that the electrolyte with an EO/Li ratio of 15:1 exhibits the highest ionic conductivity. When the EO/Li ratio is either too low or too high, the ionic conductivity decreases. As shown in Figure S3, at room temperature, the ionic conductivity is 0.053 mS cm^-1^ for EO/Li = 20:1, 0.24 mS cm^-1^ for EO/Li = 15:1, and 0.048 mS cm^-1^ for EO/Li = 10:1. Considering the overall conductivity performance, EO/Li = 15:1 was therefore selected as the optimal ratio for subsequent investigations.

**Figure S4**: Ionic conductivity and electrochemical stability windows of electrolytes with different TTE contents. (a) Room-temperature ionic conductivity. (b) Electrochemical stability window.

Following the selection of an EO/Li ratio of 15:1 as the optimal electrolyte composition, the crosslinker TTE was introduced into the system to investigate its optimal content. As shown in Figure S4a, after adding 30 mg of TTE, a significant decrease in ionic conductivity was observed, as shown in Figure S4b, with the room-temperature ionic conductivity decreasing to 0.11 mS cm^-1^. With a further reduction in the TTE content, the ionic conductivity increased to 0.18 mS cm^-1^ when 20 mg of TTE was added, and further increased to 0.23 mS cm^-1^ when the TTE content was reduced to 10 mg.

In addition, the electrochemical stability windows of the electrolytes with different TTE contents were evaluated. When the TTE content was 10 mg, 20 mg, and 30 mg, the corresponding electrochemical stability windows were 4.6 V, 4.9 V, and 5.3 V, respectively. These results indicate that the introduction of TTE effectively enhances the high-voltage tolerance of the electrolyte, while simultaneously leading to a certain reduction in ionic conductivity.

By comprehensively considering both ionic conductivity and electrochemical stability window, the TTE content was finally optimized to 20 mg for subsequent studies.

**2.8 Preparation of cathodes**

To prepare the cathode, LFP and Super C 65 (80/10 wt%) were mixed and ground for 30 minutes until a uniform powder was obtained. Then, PVDF (10 wt%) was dissolved in NMP, and the mixture was vigorously stirred for 4 h. Finally, the slurry of LiFePO_4_/Super C65/PVDF/NMP was vigorously stirred overnight. The uniform slurry was coated on aluminum foil (mass loading =1.7 mg cm^-2^) and dried under vacuum at 110 °C for 8 h.

The NCM811 electrode in the lab was prepared by mixing NCM811, Super C 65, and PVDF dissolved in NMP at a weight ratio of 8:1:1. The slurry was then cast onto aluminum foil and dried at 110 °C under vacuum for 8 h. The average loading mass of the as-prepared NCM811 cathode is approximately 1.7 mg cm^-2^.

**2.9 Cell Fabrication**

CR2016 coin cells were assembled in an argon-filled glovebox (≤ 0.1 ppm H_2_O and ≤ 0.1 ppm O_2_ ). Li||NCM811 or Li||LFP cells were assembled by using NCM811 or LFP cathode and lithium foil. The charge/discharge measurements were carried out on the NEWARE cycle test system with charge-discharge rates ranging from 0.1 to 2.0 C. at 25 ^o^C. 40 μL of polymer precursor solution was dropped onto the separator (with a thickness of 50 μm), and then the cell was assembled. The cell was heated at 60 °C for 12 h to fully polymerize the electrolyte.

3. Analytical methods

**3.1 Analysis of physicochemical properties**

Nuclear magnetic resonance (NMR) was recorded with an Advanced III 400 MHz or Advanced III 500 MHz Bruker spectrometer at 25 ^o^C. ^1^H NMR, ^13^C NMR or NOESY signals were measured relative to the signal for residual tetramethylsilane (0 ppm) in deuterated chloroform (CDCl_3_), ^7^Li, ^11^B NMR signals are reported in ppm units. Size-exclusion chromatography (SEC) measurements were performed on PL-GPC-120 system using THF as the eluant (1.0 mL min^-1^ at 40 ºC), with PLgel 10$\mu m$ MIXED-B column. The calibration was performed with polystyrene standards. Differential scanning calorimetry (DSC) measurements were performed using a Mettler-Toledo instrument at a heating rate of 10 ºC·min^-1^ under argon atmosphere. Fourier transform infrared (FT-IR) spectra were obtained from a Thermo Scientific Nicolet 6700 FT-IR instrument. X-ray photoelectron spectroscopy (XPS) was measured from a Kratos AXIS Ultra DLD instrument. Scanning electron microscope (SEM) measurements were performed on a Verios G4 instrument. Linear sweep voltammetry (LSV) and other electrochemical experiments were performed on an electrochemical analyzer/workstation (CHI 760E). The repeated charge and discharge tests were performed on a NEWARE battery test system (CT 2001A).

**3.2 Computational Details**

The theoretical calculations were performed using the Gaussian 16 suite of programs. ^[S5]^ The structures of molecules, including TPU-rotaxane (ROTAXANE), complexes of TPU-rotaxane and TFSI^-^ (ROTAXANE & TFSI^-^), PDOL, complexes of PDOL and TFSI^-^ (PDOL & TFSI^-^), BTFB, TTE, and TFSI^-^ were optimized at the B3LYP/6-31+g(d) level of theory. The structures were characterized as a local energy minimum on the potential energy surface. In the simulation, we simplified PDOL into a model compound of several structural units, simplified TPU-rotaxane into a model compound of DDI connected to an HDB and a structural unit of PTHF, and the 18C6 is appended to the urethane-N-H moiety generated from the reaction of PTHF’s terminal -OH with the -NCO group in DDI. The only difference between them and the original polymers is the degree of polymerization. We assume that the establishment of model compounds follows the same rules as the original polymers. The molecular orbital levels of the above-mentioned molecules were studied via theoretical calculations, including the highest occupied molecular orbital (HOMO) and the lowest unoccupied molecular orbital (LUMO). Besides, the color-filled iso-surface graphs of molecules were employed to visualize molecular orbitals with the aid of a visual Molecular dynamics program. ^[S6]^

The calculations of bending energy are based on the Gaussian 16 suite of programs. The optimization of molecular geometries and subsequent single-point energy calculations were performed at the B3LYP/6-31+g(d) and B3LYP/6-31+g(d, p) levels, respectively. The binding energies (E_b_) were calculated as follows.

$E_{b}=E_{complex}-\sum E_{fragment}$	S1

**3.3 Ionic conductivity measurements for polymer electrolytes**

3.3.1 *Assembly procedure for ionic conductivity measurements:*

The electrolyte between two stainless steels assembled in a CR 2016 coin cell, 40 μL of polymer precursor solution was dropped onto the separator (with a thickness of 50 μm), and then the cell was assembled. The coin cells were assembled and sealed under an argon atmosphere (O_2_ ≤ 0.01 ppm, H_2_O ≤ 0.01 ppm). The cell was heated at 60 °C for 12 h to fully polymerize the electrolyte.

3.3.2 *Calculation method for ionic conductivity measurements:*

Ionic conductivities ($\sigma$) were obtained by an alternating current (AC) impedance measurement to determine the bulk electrolyte resistances ($R_{b}^{0}$) from 20 ^o^C to 60 °C. Impedance spectroscopy measurements were carried out at a frequency range from 1 MHz to 0.1 Hz. The ionic conductivities ($\sigma$) were calculated based on Equation S2:

$\sigma=\frac{L}{R_{b}^{0}\cdot A}$	S2

The activation energies (*E_a_*) of GPEs were calculated by fitting ionic conductivity using the Arrhenius empirical equation：

$\sigma(T)=Aexp(\frac{E_{a}}{K_{B}T})$	S3

**3.4 Lithium-ion transference number measurements for polymer electrolytes**

3.4.1 *Assembly procedure for Li^+^ transference number measurements:*

The electrolyte between two lithium foils was assembled in a CR 2016 coin cell, 40 μL of polymer precursor solution was dropped onto the separator (with a thickness of 50 μm), and then the cell was assembled. The coin cell was assembled and sealed under an argon atmosphere (O_2_ ≤ 0.01 ppm, H_2_O ≤ 0.01 ppm). The cell was heated at 60 °C for 12 h to fully polymerize the electrolyte.

3.4.2 *Calculation method for Li^+^ transference number measurement:*

The Li ion transference number $t_{{Li}^{+}}$ was obtained by chronoamperometric method and EIS method. Then, the obtained Li|SPEs|Li cells were used to perform a potentiostatic polarization test at an applied voltage of 10 mV. The $t_{{Li}^{+}}$ was calculated from BruceVincent-Evans Equation:

$t_{{Li}^{+}}=\frac{I_{ss}}{I_{0}}\times\frac{（\Delta V-I_{0}\cdot R_{i}^{0}）}{（\Delta V-I_{s}\cdot R_{i}^{ss}）}$	S4

where $I_{0}$ is the initial current, $I_{ss}$ represents the steady state current, $R_{i}^{0}$ , $R_{i}^{ss}$ are the initial charge-transfer resistances and steady charge-transfer resistances respectively, $\Delta V$ is the applied polarization voltage.

**3.5 Li plating-stripping measurements for polymer electrolytes**

Assembly procedure for Li plating-stripping measurements: The electrolytes between two lithium foils were assembled in a 2016 coin cell, 40 μL of polymer precursor solution was dropped onto the separator (with a thickness of 50 μm), and then the cell was assembled. The coin cell was assembled and sealed argon atmosphere (O_2_ ≤ 0.01 ppm, H_2_O ≤ 0.01 ppm). The cells were rested at 60 °C for 12 h to fully polymerize the electrolytes. The Li||Li symmetric cells were carried out, stripping and plating test on the NEWARE cycle test system.

**3.6 SEM measurements for Li electrodes in symmetric cells**

Li electrodes were obtained from symmetric cells and washed with anhydrous THF to remove the residual polymer electrolytes in the glove box under argon. After that, Li electrodes were kept in a flask filled with argon and quickly transferred to the SEM cabin.

**3.7 Charge-discharge cycling measurements for polymer electrolytes**

The electrolytes between the lithium foil and cathode were assembled in a 2016 coin cell. 40 μL of polymer precursor solution was dropped onto the separator (with a thickness of 50 μm), and then the cell was assembled. The coin cell was assembled and sealed under an argon atmosphere (O_2_ ≤ 0.01 ppm, H_2_O ≤ 0.01 ppm). The cells were rested at 60 ^o^C for 12 h for the full polymerized of electrolytes. The Li|SPEs|cathode cells were subjected to charge-discharge cycling test on the NEWARE cycle test system with varied rates ranging from different rates.

**4. Characterization data**

**4.1 ^1^H, ^11^B and ^13^C NMR spectra of HDB**

**Figure S5.** ^1^H NMR spectrum of HDB.

**Figure S6.** ^11^B NMR spectrum of HDB.

**Figure S7.** ^13^C NMR spectrum of HDB.

**4.2 ^1^H and ^13^C NMR spectra and HRMS spectrum of TTE**

**Figure S8.** ^1^H NMR spectrum of TTE.

**Figure S9.** ^13^C NMR spectrum of TTE.

**Figure S10.** HRMS spectrum of TTE.

**4.3 ^1^H NMR spectra and NEOSY NMR spectrum of TPU-model**

**Figure S11.** ^1^H NMR spectrum of TPU-model.

**Figure S12.** NOESY spectrum of TPU-model.

**4.4 GPC spectra of TPU-rotaxane and TPU-model**

**Figure S13.** GPC spectra of TPU-rotaxane and TPU-model.

**4.5 DSC test of TPU-rotaxane and TPU-model**

**Figure S14.** The DSC curves of TPU-rotaxane and TPU-model.

**4.6 ESI tests of three kinds of electrolytes**

**Figure S15** The EIS of (a) SSNE, (b) PDOL&TTE, and (c) PDOL at different temperatures.

**4.7 Lithium-ion transference number characterizations.**

**Figure S16.** The polarization curves and transference numbers of Li|PDOL&TTE|Li cells.

**Figure S17.** The polarization curves and transference numbers of Li|PDOL|Li cells.

**4.8 TEM test of cycled cathode**

**Figure S18.** a-b)TEM images of cycled NCM811 particles with (a) SSNE, (b) PDOL & TTE. c-d) HR-TEM images of the CEI formed with (c) SSNE, (d) PDOL & TTE. e-f) The selected area electron diffraction image of cycled NCM811 cathode (e) SSNE cell, (f) PDOL & TTE cell.

**4.9 XRD measurement of cycled cathode**

**Figure S19.** XRD measurements of the NCM811 cathodes before and after cycling.

**4.10 TEM images of formed SEI**

**Figure S20.** SEI crystal plane spacing and TEM elemental analysis.

**Figure S21.** HRTEM of SEI.

**4.11 Charge and discharge curves of NCM811|SPEs|Li cell at different rate**

**Figure S22.** Charge/discharge curves of the NCM811|SSNE|Li model at 0.2 C

**Figure S23.** Charge/discharge curves of the NCM811|SSNE|Li model at 1.0 C

**4.12 Parameters of SSNE pouch cells**

**Table S1. Parameters of SSNE pouch cells.**

| **Parameters of SSNE porch cell (N/P=1.3)** | | | | | |
| --- | --- | --- | --- | --- | --- |
| **Cathode**  **(NCM811)** | **Parameter** | **Value** | **Anode**  **(Li)** | **Parameter** | **Value** |
|  | **Area capacity**  **(mAh cm^-2^)** | **3.7** |  | **Area**  **capacity**  **(mAh cm^-2^)** | **4.9** |
|  | **Area weight**  **(mg cm^-2^)** | **18.5** |  |  |  |
|  | **Size**  **(cm*cm)** | **(11*7.2)**  **(double layer)** |  |  |  |

**4.13 Charge and discharge property of LFP**

**Figure S24.** The room-temperature cycling performance of LFP||Li models with various SPEs at 0.5 C.

**Figure S****25.** The room-temperature cycling performance of LFP||Li models with various SPEs at 1.0 C.

**Figure S26.** Rate performance of the LFP|SPEs|Li cell (LFP loading: 1.7 mg cm^−2^).

**Figure S27.** Charge/discharge curves of the LFP|SSNE|Li cell at different rates.

**4.14 SSNE porch cell work in various circumstance**

**Figure S28.** A bulb powered by a charged NCM811|SSNE|Li pouch cell before and after the bending and folding scenarios.

**References:**

[S1] K. Song, W. Ye, X. Gao, H. Fang, Y. Zhang, Q. Zhang, X. Li, S. Yang, , H. Wei, and Y. Ding, *Mater. Horiz*, **2021**, 8, 216.

[S2]. B. Chen, Q. Xu, Z. Huang, Y. Zhao, S. Chen, X. Xu, *J. Power Sources*, **2016**, *331*, 322.

[S3]. D. Sharon, P. Bennington, M. A. Webb, C. Deng, J. J. de Pablo, S. N. Patel, P. F. Nealey, *J. Am. Chem. Soc.*, **2021**, *143*, 3180.

[S4]. H. Wang, P. Wang, G. Shi, *J. Phys. Chem. Solids*, **2024**, *193*, 112190.

[S5] M. Frisch, G. Trucks, H. Schlegel, G. Scuseria, M. Robb, J. Cheeseman, G. Scalmani, V. Barone, G. Petersson, H. Nakatsuji, *Gaussian16 (Revision A. 03),* **2016**.

[S6] W. Humphrey, A. Dalke, K. Schulten, *J. Mol. Graphics* **1996**, *14*, 33.
